# Incidence and Duration of Sick Leave Due to Work-Related Musculoskeletal Disorders in the Accommodation and Food Services Activities Sector in Slovenia: A Nationwide 5-Year Observational Study

**DOI:** 10.3390/ijerph20043133

**Published:** 2023-02-10

**Authors:** Dorjana Zerbo Šporin, Žiga Kozinc, Ticijana Prijon, Nejc Šarabon

**Affiliations:** 1 Faculty of Health Sciences, University of Primorska, SI-6310 Izola, Slovenia; 2 Andrej Marušič Institute, University of Primorska, SI-6000 Koper, Slovenia; 3 National Institute of Public Health, SI-1000 Ljubljana, Slovenia; 4 Human Health Department, InnoRenew CoE, SI-6310 Izola, Slovenia; 5 Laboratory for Motor Control and Motor Behavior, S2P—Science to Practice, Ltd., SI-1000 Ljubljana, Slovenia

**Keywords:** absenteeism, accommodation and food service, epidemiology, musculoskeletal disorders, workplace

## Abstract

Objectives: The aim of this study was to analyze Slovenian data on sick leave (SL) due to the most common work-related musculoskeletal disorders (MSDs) among workers in sector I “accommodation and food services activities” from the NACE Rev2 classification. Methods: We investigated both SL incidence (i.e., number of cases) and disease severity (i.e., average SL duration) by body site, gender, age and divisions within the sector. In addition, trends in SL data (difference between 2015 and 2019) were analyzed. The effect of age group, gender and division was also assessed with relative risk (RR). RESULTS: Female gender was associated with higher risk of MSDs in young (RR = 1.91 (1.53–2.43)) and older (RR = 2.24 (1.90–2.65)) subgroups. Older age was associated with greater SL incidence and longer SL duration, regardless of gender and division within sector I. This was also reflected in relative risk calculations between older and younger groups (females: RR = 4.43; CI = 3.75–5.01; *p* < 0.001; males: RR = 3.71; CI = 2.89–4.77; *p* < 0.001). Low back disorders were the most frequent cause for SL, while lower limb disorders tended to cause the longest average SL. The SL durations were similar across divisions within the sector, while the incidence rate tended to be higher in the “accommodation” than the “food and beverage services” division. Conclusions: Special attention needs to be paid to reducing the risk of low back disorders, which are by far the most common cause of SL, and lower limb disorders, which cause the longest SL. We recommend the implementation of countermeasures that focus on early detection and rapid treatment/recovery of MSDs in older workers.

## 1. Introduction

Musculoskeletal disorders (MSDs) are still the most common work-related health issue in the European Union (EU). MSDs have consequences for workers, businesses and society, including absenteeism from work [1]. In Slovenia, absence from work due to work-related MSDs accounts for 15% of total sickness absence and for 25% of long-term sickness absence. Although all workers can be affected by MSDs, the prevalence and the specificity of MSDs vary considerably by occupation and work sector. Significant differences have also been found between occupational sectors in the prevalence of obesity and other factors that may contribute to the development of MSDs [2]. Therefore, certain sectors may need additional attention to reduce the prevalence of MSDs.

The accommodation and food services activities sector (NACE Rev2 Sector I) is an important job creator, employing 8.3% of the EU workforce in 2019 [3]. In Slovenia, the number of employees in this growing sector increased by 14% from 2015 to 2021, with more women (60%) employed than men [4]. This sector is associated with a high risk of developing musculoskeletal disorders, but the figures vary considerably in different studies. For example, a study of over 900 participants in Taiwan reported that 84% of restaurant workers suffered from work-related musculoskeletal disorders, with the shoulder being the most common and the low back the most severe [5]. Another study conducted in the United States confirmed the high prevalence of low back and shoulder disorders, but reported that the annual prevalence was only 42% [6]. In restaurant businesses, women appear to be at slightly higher risk than men, and certain occupations within the industry may be associated with particularly high risk [7]. Among hotel workers, the overall prevalence of MSDs is generally similar for men and women, but certain departments and occupations may be associated with a gender-specific increased risk of MSDs [8]. Section I workers are highly exposed to physical factors associated with MSDs. Poor job design, standing for long periods, working in awkward positions, lifting and moving loads, and strenuous and repetitive tasks can lead to MSDs [1,9,10,11]. These workers are not only exposed to physical strain, but also to psychosocial risks. Constant customer contact, long and unusual working hours, and time pressure are examples of the risk factors they face [10] that can exacerbate MSDs [12].

Our previous study also showed that workers in the “accommodation and food service activities” sector in Slovenia are at high risk for MSDs. For example, female workers from this sector aged 45.0–64.9 years have on average the fourth highest incidence of work-related low back disorders [13]. Therefore, the aim of this study was to determine the gender-, age-, and site-specific incidence and severity of work-related MSDs in the “accommodation and food services activities” sector as a whole and in its divisions from 2015 to 2019. By using a nationwide database of MSD-related sick leave (SL), this study helps prioritize problems for further application of specific MSD prevention strategies in sector I. To our knowledge, no study has yet been published that has analyzed such comprehensive MSD-related SL data in the “accommodation and food services activities” sector.

## 2. Materials and Methods

### 2.1. Study Population and Data Collection

We analyzed Slovenian national data for SL determinants due to the most common work-related MSDs in the NACE Rev2 sector I “accommodation and food services activities”. The analysis was made for the period 2015 to 2019 by gender, body region and two age groups: 20.0–44.9 years (20–44 y) and 45.0–64.9 years (45–65 y). Results are presented for both sector I as whole and separately for its divisions.

Data regarding SL represent an important source of information on the health status of the working population. The data for this study were collected by the Slovenian Institute of Public Health (NIJZ). All data were anonymized at all stages of the study. The study does not contain data that could be linked to an individual person. The NIJZ collects, analyzes and disseminates data on SL of employed and self-employed workers who are enlisted in the compulsory health insurance in Slovenia. Data collection covers the work force in all NACE Rev2 economic activities. Data collection has a legal basis in the Health Care Databases Act (ZZPPZ-Ur. l. RS 65/00, database NIJZ3) and Personal Data Protection Act (ZVOP-1—Ur. L. RS 94/07). Article 17 in the Personal Data Protection Act considers scientific research for historical or statistical purposes as a lawful processing operation and therefore provides a legal basis for further processing. The source of data is the Certificate of justified abstinence from work due to health conditions (eBOL). The data are obtained from health care providers. The distribution of the data in this paper was also approved by the Ethical Committee of the National Institute of Public Health (Approval number: 6310-1/2021-35 (241). The study was conducted in accordance with the guidelines of the Declaration of Helsinki.

### 2.2. Classification of Economic Activities

According to the NACE Rev 2—Statistical classification of economic activities in the European Community—the activities are separated into 21 sectors. The NACE Rev 2 sector I, “accommodation and food services activities”, contains two divisions: “accommodation” (No. 55) and “food and beverage service activities” (No. 56). Accommodation activities include the provision of short-stay accommodation for visitors and other travelers. Food and beverage service activities provide complete meals or drinks fit for immediate consumption [11].

### 2.3. Data Analysis and Outcome Measures

For the analysis, we received anonymous data as numbers representing a body-region-specific SL determinant by gender and age in “accommodation and food services activities” and it’s divisions. The average values for SL determinants from 2015 until 2019 and by single year were used for analysis. First, we analyzed (i) the incidence rate, as the number of SL cases per 100 employees in one year (SL incidence) and (ii) the severity of MSDs, expressed as the average duration of one absence from work due to a health condition (SL duration). The number of SL cases is considered to be a number of completed SL due to MSDs in a calendar year (1 January–31 December) regardless of when the SL started. The most common work-related MSDs (according to the ICD-10-AM classification) by body region (upper back, low back, shoulders, elbow, hand and wrist, hip, knee, and ankle) included in the study are listed in Table 1. For total SL incidence rate (all MSDs combined), we also calculated the relative risk (RR) with 95% confidence intervals (CI) to assess the risk difference between age groups, gender and divisions.

## 3. Results

### 3.1. Workers in “Accommodation and Food Services Activities”

The number of employees in the sector “accommodation and food services activities” sector I, has increased from 32,526 in 2015 to 37,928 in 2019. They account for 4.2% of the Slovenian workforce. There are more females than males employed in this sector (approximately 1.5:1 ratio), with the number of females ranging from 19,452 to 22,529 and the number of males ranging from 13,074 to 15,399 in 2015–2019. In addition, there are about 1.8 times more workers in the 20–44 age category (24,485) than in the 45–65 category (13,443). Most workers (71%) are in the “food and beverage services activities” and 29% are in the “accommodation” division. For more details, see Table 2, which shows the exact data on sector I employment in 2019 [4].

### 3.2. Sick Leave Incidence and Severity of the Most Common Work-Related Musculoskeletal Disorders in Sector I by Age, Gender and Body Region

**Figure 1:**
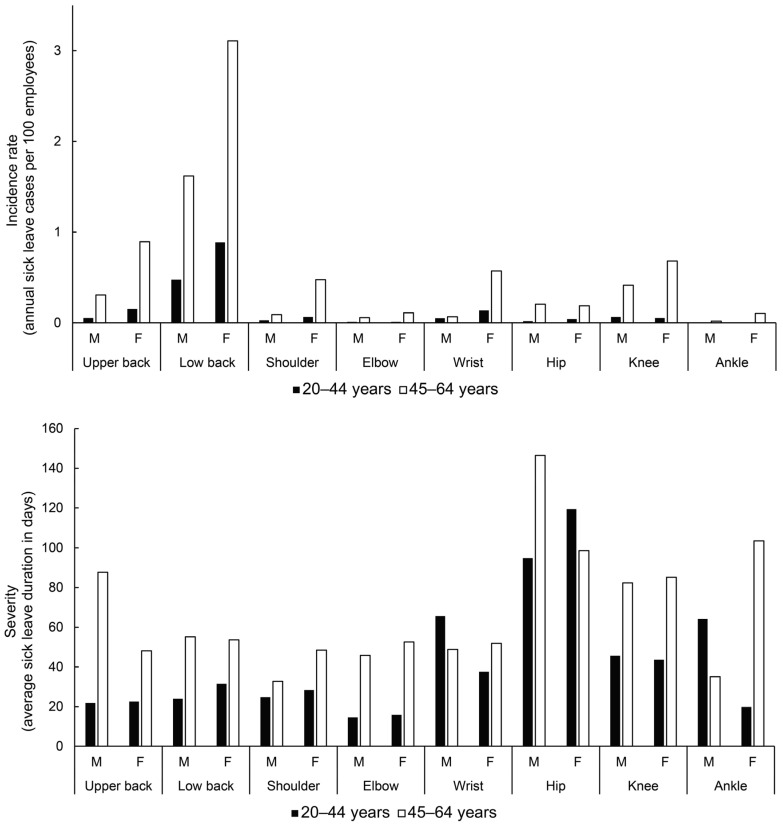
Average sick leave determinants due to work-related musculoskeletal disorders (2015–2019) in sector I by age group, gender and body region. M—male; F—female.

Figure 1 shows the average SL incidence and SL duration of data from 2015 to 2019 for all of sector I, separated by age, gender, and body region. The top chart depicts the incidence rate as the number of SL cases per 100 employees in a year. The low back region was by far the most problematic in both age groups and genders, ranging from 0.47 cases in males in the younger group to 3.10 cases in females in the older group. In addition to low back disorders, which appear to be prevalent in both genders and age groups, the older female group also showed relatively high numbers of SL due to upper back, shoulder, wrist and knee MSDs. The older group had a higher SL incidence of cases consistently across the body regions.

The bottom chart shows the data on the average sick leave duration. The data showed high variability across the body regions (total range = 14.5 to 146.5 days). In the back and upper limbs, the older group generally had a longer SL duration (range = 32.7 to 87.7 cases) than the younger group (range = 14.5 to 31.5 cases), with the exception of the wrist, where there was little difference between age groups. SLs due to hip disorders had the highest severity, with average SL duration ranging from 94.8 to 146.5 days. There were no major differences between genders; in the older group, males had more severe upper back disorders (SL duration = 87.7 days, compared with 48.1 days in females), while older group females had longer SLs due to ankle disorders (SL duration = 103.5 days, compared with 19.8 days in males).

### 3.3. Trends in Sick Leave Due to the Most Common Work-Related Musculoskeletal Disorders in Sector I from 2015 to 2019

**Figure 2:**
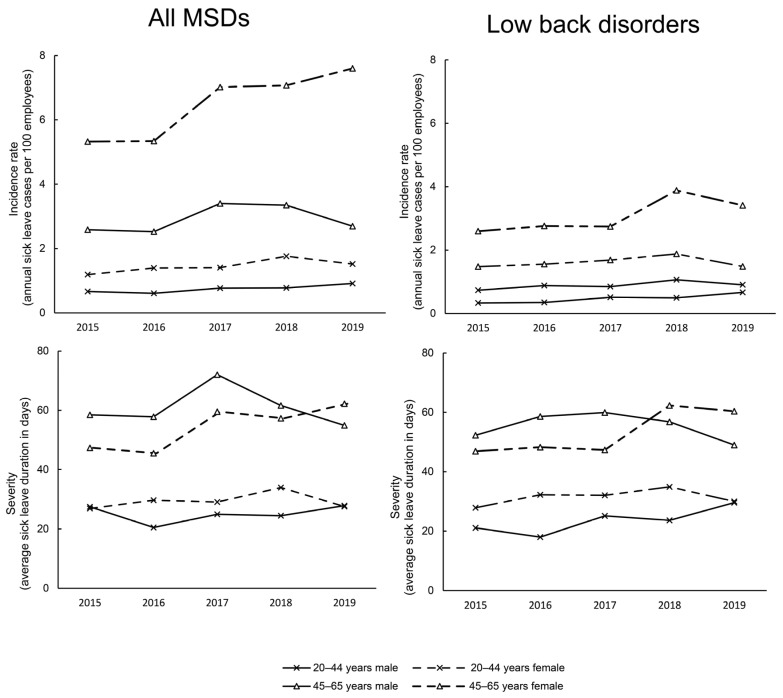
Trends from 2015 to 2019: incidence of musculoskeletal disorder cases and their severity for all body regions combined.

Figure 2 shows the SL determinants between 2015 and 2019 for all body regions combined. The SL incidence (i.e., the number of cases per 100 employees in one year, top chart) in the younger subgroup was lower in males (0.61 to 0.91 cases) than females (1.19 to 1.75 cases). Accordingly, female gender increased the risk of SLs for 92% compared to males (RR = 1.92; CI = 1.53–2.43; *p* < 0.001) in the young subgroup. In the older males, the SL incidence range from 2.52 to 3.39 cases. Among older females, the numbers were even higher, with 5.32 cases in 2015, followed by an increasing trend toward 7.59 cases in 2019. Female gender increased the risk of SLs for 124% compared to males (RR = 2.24; CI = 1.90–2.65; *p* < 0.001) in the older subgroup. Older groups had noticeably higher incidence rate, which is also reflected in relative risk calculations (females: RR = 4.43; CI = 3.75–5.01; *p* < 0.001; males: RR = 3.71; CI = 2.89–4.77; *p* < 0.001).

Data on SL duration (i.e., average duration of SL) show consistent values over the years, with males consistently showing slightly shorter duration (20.5–27.8 days) than females (26.9–33.9 days). SL duration, like SL incidence, appears to be increasing in the older female group (2015 = 47.3 days; 2019 = 62.2 days). Males had even larger values in 2015–2017 (difference ~ 10–11 days), but decreased after 2017 and ended up at lower values than females in 2019 (54.8 days).

### 3.4. Sick Leave Incidence and Severity of the Most Common Work-Related Musculoskeletal Disorders in Sector I Divisions

**Figure 3:**
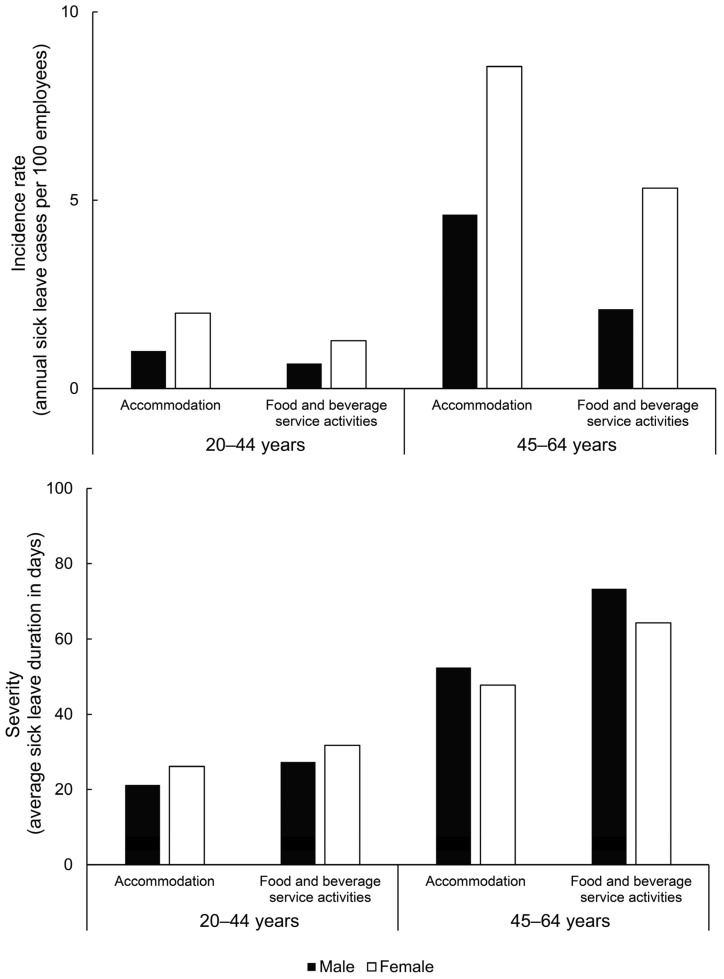
Sick leave determinants due to musculoskeletal disorders per divisions of sector I.

The analysis of SL incidence by division shows that age has a greater impact than divisions (Figure 3, top chart). The SL incidence is higher in females across all age groups and divisions. The “accommodation” division presented with higher numbers than “food and beverage services” in young male (1.0 vs. 0.67 cases), young female (2.0 vs. 1.27 cases), older male (4.62 vs. 2.11 cases) and older female groups (5.32 vs. 8.55 cases). In young males, the risk was not different between the divisions (RR = 1.51; CI = 0.94–2.39; *p* = 0.081), but it was statistically significantly higher in “accommodation” than “food and beverage services” for young females (RR = 1.57; CI = 1.18–2.08; *p* = 0.002), older males (RR = 2.19; CI = 1.58–3.04; *p* < 0.001) and older females (RR = 1.60; CI = 1.36–1.88; *p* < 0.001).

Regarding the severity of SL (Figure 3, bottom chart), the division had a smaller effect. Again, age was the most important determinant (range = 21.2–31.8 days in younger groups; 47.8–73.4 in older groups). The “food and beverage services” division had higher SL duration than the “accommodation” division, with the most evident difference in older groups (73.4 vs. 52.4 days for older males; 64.3 vs. 47.8 days for older females).

## 4. Discussion

Sector I “accommodation and food service activities” of the classification NACE Rev2 seems to require additional attention and specific countermeasures to reduce MSDs [7,10,11]. This industry contributes significantly to employment and the economy in the EU [14]. From 2015 to 2019, the number of employees in sector I in Slovenia increased by 17% [4]. The present study is therefore particularly important because a comprehensive, nationwide database allows a detailed assessment of the gender-, age-, and body region-specific incidence and severity of the most common work-related MSDs in sector I and its divisions. Our findings could help identify risk groups for musculoskeletal disorders in the “accommodation and food service activities” sector and develop better prevention and management strategies for MSDs.

### 4.1. Body-Site-Specific MSDs

Low back disorders (LBDs) were by far the most common (SL incidence) work-related MSDs in both gender and age groups (Figure 1). This finding is consistent with the results of previous studies, reporting that workers in the hotel [15,16] and restaurant [17,18] industries suffered mainly from low back pain. Although the most commonly affected body region was the low back, the hip, including osteoarthritis and other cartilage disorders (Table 1), was associated with the longest mean SL duration. For LBDs, sick leave is usually short, with an average duration of 26 days. For hip MSDs, sick leave is generally longer, especially for hip osteoarthritis, where the average duration is 117 days [19]. Figure 1 shows an interesting MSD pattern: on the left, a relatively high SL incidence and short SL duration for the upper limbs and trunk, while on the right, the relatively low SL incidence and long SL duration for the lower limbs. An exception on the left is the wrist in both genders and the upper back in older male workers, who on average have a difficult course of MSDs with relatively low SL incidence. Compared to workers suffering from upper limb and trunk MSDs, workers with lower limb MSDs are more likely to have symptoms lasting longer than two months [20]. Park et al. [21] found that, in the “hotel and restaurants” sector, workers are highly exposed to ergonomic risk factors such as awkward postures, handling loads, and prolonged standing/walking. These tasks are associated with musculoskeletal disorders of the lower limbs but also with MSDs of the upper limbs and trunk [22]. Efforts to reduce exposure to these tasks could help reduce the overall risk of MSDs in the “accommodation and food service activities” sector. While LBDs are the most prevalent, the high duration of SLs due to lower limb disorders is also a cause for concern. Reducing the prevalence of lower limb disorders would likely reduce the healthcare costs; for instance, osteoarthritis costs for newly diagnosed patients are almost 7000 USD per year according to the data from United States [23]. When surgery is needed, up to 40,000 USD is spent on each patient [24]. In summary, reducing the incidence and prevalence of both LBDs and lower limb disorders would benefit both the workers and the healthcare system.

### 4.2. Gender-Specific MSDs

Our study revealed (Figure 1) a gender difference in work-related MSDs in sector I. Similar to other studies [7,8], MSDs occur more frequently in female workers in almost all body regions. The observed gender difference was particularly pronounced in older workers. Of particular concern, the incidence of MSDs in older females increased by 42% between 2015 and 2019 (Figure 2). Their average SL duration is also increasing, exceeding that of older men in 2019, for whom MSD-related SL determinants are actually declining. LBDs appear to be an important factor in this observed trend, as disabling LBDs are significantly associated with female gender [25]. Therefore, gender differences should be considered in the prevention of MSDs in sector I. Particular attention should be paid to reducing the risk of MSDs, especially LBDs, among female workers, who represent 60% of the workforce in the “accommodation and food service activities” sector. Given the increasing severity of MSDs in older females, early diagnosis and prompt treatment of these conditions are also important. Further studies are needed to determine the causes of gender differences in sector I MSDs.

### 4.3. Age-Specific MSDs

Consistent with previous publications [12,26], older workers in sector I are more susceptible to work-related MSDs than younger workers in all body regions (Figure 1). Age is not an independent risk factor for MSDs in the workplace [27], because as workers age, the mismatch between job demands and functional capacity increases, leading to musculoskeletal injuries [26]. We observed the unfavorable relationship as higher incidence/longer duration of MSDs mainly in older workers. This means that older workers generally take longer to recover from MSDs than younger workers for the same condition [26]. The exceptions were the hips in females and the wrists and ankles in males (Figure 1). Apparently, in these cases, only a few severe MSDs drive up the average SL duration in younger workers. For example, our database shows that SL was associated with hip osteoarthritis or other cartilage disorders in only five cases of young female workers, for which they took an average of 116 days of sick leave. As shown in the present study (Figure 1), hip osteoarthritis is more common in the older population [28]. Patients with advanced osteoarthritis undergo hip replacement to relieve pain and improve function [29]. Osteoarthritis is the most common indication for hip replacement surgeries [30]. In such cases, recovery is long-lasting, with clinically significant improvements in the 6- to 12-month recovery period [31], which could explain why the hip region exhibited the longest SL duration. We also found an age difference in MSDs trends in 2015–2019 for sector I (Figure 2). While the trend for MSD-related SL was stable in younger females, it worsened over the years in older females. This is different for males. A stable trend was observed for younger workers, while a positive trend was observed for older workers in the form of a decrease in MSD-related SL. Based on the obtained results, we advise that measures to prevent work-related MSDs in sector I should be implemented for all workers. In particular, for older workers, it is useful to focus on early detection and appropriate treatment of MSDs.

### 4.4. Division-Specific MSDs

It appears that in sector I, older age contributed more to the incidence and severity of the most common work-related MSDs than occupational exposure in each division (Figure 3). This finding and the results of other studies support the need for workplace-specific risk surveillance to ensure that all factors contributing to MSD risk can be closely monitored, regardless of age [32]. It is also important to consider that susceptibility to MSDs is related to the difference between job demands and the functional capacity of the worker rather than to age [26]. Age and gender appear to be the most important determinants of SL. Nevertheless, we found somewhat different SL determinants of work-related MSDs in the different divisions of sector I (Figure 3). The incidence was higher in the “accommodation” division, but more severe courses of MSDs were found in the “food and beverage services” division, which was particularly noticeable in older workers. Therefore, it is possible that occupational exposure of workers in the “food and beverage services” division results in more severe work-related MSDs than in the “accommodation” division. Peng et al. [7], who reported population-based data in the “food and beverage industry,” showed an incidence rate of 4.71 MSDs per 100 persons in a year. In our study, we found MSD-related cases ranged from 0.67 per 100 young male workers to 8.55 per 100 older female workers in a year. Work in the food service industry is associated with several physical risk factors, such as repetitive lifting, carrying, prolonged standing, bending, twisting, repetitive movements, awkward postures and overexertion, which contribute to MSDs [33,34]. Nevertheless, it seems that age (and to some extent gender) is the most important determinant of SL incidence and duration. Therefore, all divisions of the sector should be fully considered in the prevention and treatment of MSDs.

### 4.5. Limitations

The major limitation of the study is the lack of data on specific workplaces within sector I and its divisions. At least a small proportion of workers were engaged in administrative tasks, and it may be that this proportion varies between age groups and genders. Rather than dividing sectors into divisions, further research should consider classifying individuals by job type to identify which individuals need special attention in terms of MSD prevention. We also need to be cautious about generalizing the results to other geographic locations.

## 5. Conclusions

This study provides a nationwide overview of SL incidence and SL duration of the most common work-related MSDs in sector I “accommodation and food service activities” from the classification NACE Rev2. Special attention should be paid to reducing the risk of low back disorders, which are by far the most common cause for MSD-related sick leave, and lower limb disorders, which cause the longest SL. Older age has been associated with higher SL incidence and longer SL duration. Therefore, we recommend countermeasures that focus on early detection and rapid treatment/recovery of MSDs in older workers. Prevention strategies should consider the role of gender and age, while the influence of division on SL risk is likely to be small.

## Figures and Tables

**Table 1:** List of the most common work-related musculoskeletal disorders included in the study by body region.

Body Region	Included Musculoskeletal Disorders
Upper back	cervical disk disorders (M50.0–M50.9), cervicocranial syndrome (M53.0), cervicobrachial syndrome (M53.1), cervicalgia (M54.2)
Low back	other intervertebral disk disorders (M51.0–M51.9), sciatica (M54.3), lumbago with sciatica (M54.4), low back pain (M54.5)
Shoulder	adhesive capsulitis of shoulder (M75.0), rotator cuff tear or rupture, not specified as traumatic (M75.1), bicipital tendinitis (M75.2), calcific tendinitis of shoulder (M75.3), impingement syndrome of shoulder (M75.4), bursitis of shoulder (M75.5), other shoulder lesions (M75.8), shoulder lesion, unspecified (M75.9)
Elbow	medial epicondylitis (M77.0), lateral epicondylitis (M77.1), olecranon bursitis (M70.2), other bursitis of elbow (M70.3)
Hand and wrist	osteoarthritis of first carpometacarpal joint (M18.0–M18.9), radial styloid tenosynovitis (de Quervain) (M65.4), crepitant synovitis of hand and wrist (M70.0), peri arthritis of wrist (M77.2), carpal tunnel syndrome (G56.0)
Hip	osteoarthritis of hip (M16.0–M16.9), other articular cartilage disorders of hip (M24.15)
Knee	osteoarthritis of knee (M17.0–M17.9), internal derangement of knee (M23.0–23.9), prepatellar bursitis (M70.4), other bursitis of knee (M70.5), synovial cyst of popliteal space (Baker) (M71.2)
Ankle	primary osteoarthritis of ankle and food (M19.07), secondary osteoarthritis of ankle and (M19.27), other and unspecified osteoarthritis (M19.)

**Table 2:** The number of employees in sector I in 2019 by age, gender and divisions.

Divisions	20–44 Years			45–65 Years		
	M	F	Total	M	F	Total
Accommodation	2604	3750	6354	1557	3140	4697
Food and beverage service activities	7968	10,163	18,131	3270	5476	8746

M—male; F—female.

## Data Availability

All data are presented within the manuscript.
